# ARF1 and SAR1 GTPases in Endomembrane Trafficking in Plants

**DOI:** 10.3390/ijms140918181

**Published:** 2013-09-05

**Authors:** Birsen Cevher-Keskin

**Affiliations:** Plant Molecular Biology Laboratory, Genetic Engineering and Biotechnology Institute, Marmara Research Center, The Scientific and Technical Research Council of Turkey, TUBITAK, P.O. Box: 21, Gebze 41470, Kocaeli, Turkey; E-Mail: birsen.keskin@tubitak.gov.tr; Tel.: +90-262-677-3313 (ext. 123); Fax: +90-262-677-646-3929

**Keywords:** GTPases, vesicular trafficking, ARF1, SAR1, COPI, COPII

## Abstract

Small GTPases largely control membrane traffic, which is essential for the survival of all eukaryotes. Among the small GTP-binding proteins, ARF1 (ADP-ribosylation factor 1) and SAR1 (Secretion-Associated RAS super family 1) are commonly conserved among all eukaryotes with respect to both their functional and sequential characteristics. The ARF1 and SAR1 GTP-binding proteins are involved in the formation and budding of vesicles throughout plant endomembrane systems. ARF1 has been shown to play a critical role in COPI (Coat Protein Complex I)-mediated retrograde trafficking in eukaryotic systems, whereas SAR1 GTPases are involved in intracellular COPII-mediated protein trafficking from the ER to the Golgi apparatus. This review offers a summary of vesicular trafficking with an emphasis on the ARF1 and SAR1 expression patterns at early growth stages and in the de-etiolation process.

## 1. Introduction

The functional organization of eukaryotic cells requires the exchange of proteins, lipids, and polysaccharides between membrane compartments through transport intermediates. Transport from one compartment of this pathway to another is mediated by vesicular carriers, which are formed by the controlled assembly of coat protein complexes (COPs) on donor organelles. The activation of small GTPases is essential for vesicle formation from a donor membrane. In eukaryotic cells, small GTP-binding proteins comprise the largest family of signaling proteins. Four main subfamilies have been identified in plants; (i) ARF/SAR; (ii) RAB; (iii) ROP (Rho-like proteins in plants); and (iv) RAN [1–3].

Over the evolution of eukaryotic organisms, the conservation of GTPases implies their significance in cellular signaling processes [3–5]. Previous studies have shown that the steps in endomembrane trafficking—from the endoplasmic reticulum (ER) to the Golgi, intra-Golgi, post-Golgi, and endosome—are mediated by subfamilies of the RAB and ARF GTPases in yeast (*Saccharomyces cerevisiae*) and mammalian systems [6].

**Figure 1:**
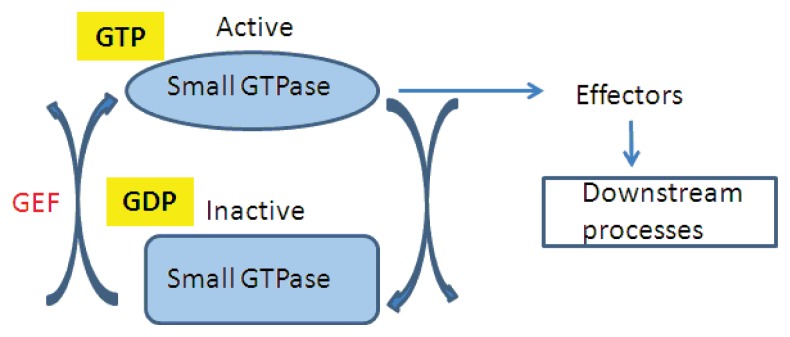
GTPases cycle between an active GTP-bound state and an inactive GDP-bound state. Guanine Exchange Factors (GEFs) activate GTPases, which in turn interact with specific effectors to mediate downstream pathways. The intrinsic GTPase activity of these small G proteins is stimulated by GAPs, which accelerate the inactivation of the regulatory activity of the GTPases.

Small GTPases serve as molecular switches that transduce signals by exchanging between the GTP- and GDP-bound conditions. Guanine-nucleotide exchange factors (GEFs), GDP dissociation inhibitors (GDIs), and GTPase-activating proteins are regulators of small GTP-binding proteins (Figure 1).

The GEFs activate small GTPases, which in turn interact with specific effectors to mediate downstream pathways. GAPs stimulate the intrinsic GTPase activity, thereby accelerating the inactivation of the GTPases’ regulatory activity.

GEFs convert the GDP-bound inactive form of the GTPases to the GTP-bound active form by stimulating the dissociation of GDP from the GDP-bound form. In the “active” state, the GTP-bound GTPases interact with various downstream effector proteins that execute diverse cellular functions. GTPases are inactivated through either the intrinsic capability of the GTPase to hydrolyze GTP to GDP+Pi or an interaction with another protein group, the GTPase-activating proteins (GAPs). These proteins catalyze the hydrolytic activity of GTPases, which then return to the inactive state GDP-bound state [2]. The improvement of fluorescent protein-labeled GTPases and cargo molecules has accelerated the assignment of subcellular locations for these proteins within the endomembrane system.

This review focuses on plant ARF1 and SAR1 GTPases and on the role of these small GTP-binding proteins in the regulation of membrane trafficking and cell polarity.

## 2. ARF1 (ADP-Ribosylation Factor 1) and SAR1 (Secretion-Associated RAS Super Family 1) GTPases

Protein coats are classified into three types for transport vesicles: COPI, COPII, and clathrin coats [7,8]. Arrangement of these coats is attributed to two distinct subsets of the ras superfamily. ARF GTPases recruit COPI and clathrin protein coats to transport vesicles. The SAR1p GTPases, a specific subset of the ARF GTPase family, recruit COPII coats.

### 2.1. ARF1

Similar to other small GTPases, ARF GTPases cycle between an active, membrane-bound form when associated with GTP and an inactive, predominantly cytosolic form when bound to GDP. In both animal and plant cells, a large number of ARFGAPs have been identified and categorized into several subfamilies [3]. In the mammalian system, Arfs are divided into three classes and express six isoforms, namely, Arf1-6 (with Arf2 being absent in human).

On the other hand, of the 12 ARF isoforms, ARF1 is targeted to the Golgi and post-Golgi structures in plant cells. ARF1 facilitates ER-to-Golgi transport and Golgi-derived transport to the plasma membrane, depending on the COPI vesicle coat protein components [2,7]. The large number of ARF in plants offers the possibility for highly regulated vesicle trafficking [2]. ARF1 and ARFB, which shows significant homology to mammalian ARF6 have been the most widely characterized. In plants, ARF1 has been shown to localize to Golgi and endosomes, and regulates vesicle trafficking, cell proliferation, cell elongation and fertility whereas ARF6 is associated with plasma membrane and involved in receptor endocytosis and actin remodeling [7,8]. Furthermore, ARF6 is overexpressed in highly invasive breast cancer cells and plays an essential role during invasion through ERK signaling [9,10]. It was demonstrated that ARF1 overexpression might contribute to a poor cancer prognosis. The ARF1 protein can be used as a prognostic marker for gastric cancer [11].

#### 2.1.1. COPI and Retrograde Transport

The COPI coatomer is a protein complex composed of seven subunits (α, β, β′, γ, δ, ɛ, and ζ-COP).

It represents approximately 0.2% of soluble cytosolic protein [12]. Arabidopsis has single genes for γ-COP and δ-COP and multiple genes for the other COPI subunits [13]. COPI coatomer forms a coat around vesicles budding from the Golgi.

COPI proteins can be purified from the cytosol, indicating their roles as unassembled precursors of COPI vesicles. Two differently sized COPI vesicles have been recognized in *Arabidopsis* by multiparameter electron tomography analysis [14]. Whereas COPIa coats retrograde transport vesicles, COPIb vesicles are restricted to *medial*- and *trans*-cisternae and most likely are responsible for retrograde transport within the Golgi stack. The multiple copies of COPI suggest the existence of different classes of COPI vesicles in plants. The *Arabidopsis* genome encodes two or more isoforms of each COPI protein [2]. In plants, γ- and ɛ-COP proteins have been identified and localized in the Golgi by immunolabeling experiments [10,14].

In the early secretory pathway, COPI vesicles have been suggested to mediate different transport steps, including ER-to-Golgi intermediate compartment transport, Golgi transport, and/or intra-Golgi transport (anterograde transport and/or retrograde transport from the Golgi to the ER) [15,16]. It was assumed that two types of COPI-coated vesicles form at the Golgi apparatus level containing anterograde or retrograde cargo (KDEL receptor) and low amounts of Golgi enzymes [17]. Martinez-Menarguez and colleagues [18] demonstrated that retrograde trafficking-directed Golgi enzymes are more likely than anterograde cargo to be found in peri-Golgi vesicles by double-labeling experiments in the mammalian system. It was also established in the same system that COPI proteins are involved in transport along the endocytic pathway [19,20].

During the selective transport of vesicles, the coat proteins must distinguish between cargo and resident proteins of the donor organelle. In intracellular transport, cargo transmembrane protein sorting at each step depends on the specific interaction of certain motifs (sorting signals) in their cytoplasmic tails with the correct coat proteins [21]. A cytosolic dilysine motif is crucial for the ER localization of type I membrane proteins in yeast and mammalian cells [22]. The two lysine residues must be in the −3, −4 (KKXX) or −3, −5 (KXKXX) positions relative to the carboxy (C) terminus [22]. For ER localization, the lysine residue at the −3 position is the most critical residue [23]. In mammals, lysine residue mutations within the KKXX motif lead to the expression of reporter proteins at the cell surface [22]. In contrast, the same mutation leads to vacuolar transfer in yeast [24].

The p24 proteins have been suggested to function in Golgi-to-ER retrograde transport, as they contain cargo receptors on their luminal side and coatomer and/or ARF1 receptors on their cytoplasmic side in mammalian cells [25–28]. COPI is necessary for recycling p24 proteins to the ER from the Golgi apparatus [29].

The p24 proteins have classical dilysine motifs at the −3 and −4 position, which bind COPI and mediate Golgi-to-ER retrograde transport. In general, these proteins are only found in the ER. Langhans *et al*. [29] found that when the ARF1 (Q71L) mutant was expressed at low levels in tobacco mesophyll protoplasts, there was an accumulation of wt p24 in the Golgi apparatus as well. These results confirm that the COPI-recycling mechanism can efficiently function in plants. p24 mutants deficient in the COPI-binding dilysine motif are transported to the PVC and vacuole [29].

ARF1 is present in its GDP form in the cytosol and is recruited to the surface of Golgi membranes by a GEF. A SEC7-type GEF promotes the binding of GTP to ARF1. This process can be inhibited by the fungal metabolite Brefeldin A (BFA) in mammalian cells [30]. In animal cells, the GDP-bound form of ARF1 interacts with p24 cytosolic tails [31]. The activation of cytosolic ARF1 initiates COPI biogenesis. The GTP/GDP exchange follows a conformational change in ARF1 that may cause its dissociation from p24 cytosolic tails [31]. The GTP-bound form of ARF1 interacts with coatomer, which can also interact directly with the cytosolic tails of p24. Therefore, the p24 cytosolic tail can interact both with ARF1 and coatomer [31].

Donaldson *et al*. 1992 [32] demonstrated that binding and conformational changes of the GTPase leads to interaction with the *N*-terminal myristoyl-anchor and an amphipathic helix in the mammalian system [33]. The ARF1 and SEC7 domain protein interaction can be facilitated and altered by additional lipid-protein interactions that activate the proteins next to the membrane surface [34]. In mammalian and plant cells, even though p23 was initially assumed to be the ARF1/coatomer receptor, it appears that other p24 proteins may also interact with ARF1 and/or coatomer [35,36].

The p23 and other p24 family members have been suggested to have structural and morphogenic roles in the organization and/or biogenesis of the Golgi complex [37]. The p25-alpha (2) p24 proteins have been shown to be involved in the formation of ER-Golgi intermediate compartment (ERGIC)/vesicular tubular clusters and ER exit sites [38]. In retrograde transport, it was demonstrated that p25 may function as an anchor for the p24 proteins [39]. In *Arabidopsis*, up to 11 different p24 family members proteins have been identified. The plant p24 proteins contain signals for binding both COPII and COPI subunits. These proteins appear to bind COPI (retrograde transport) with higher affinity than COPII (anterograde transport) [36].

The dilysine motif in the cytosolic tail of the *Arabidopsis* p24 protein (Atp24) is required both for binding of coatomer subunits and ARF1 *in vitro* [36]. In Arabidopsis, it was also shown that the dihydrophobic (FF or YF) motif in the −7 and −8 positions is necessary and sufficient for COPII binding, especially for the SEC23/24p subunits [36]. Furthermore, this motif co-operates strongly with the dilysine motif in ARF1 and coatomer binding [36,40].

It has been shown that ARF1 plays an essential role in normal cell growth [41]. Xu and Scheres [42] demonstrated that ARF1 function is essential for plant development and cell polarity and is ubiquitously expressed in all organs of *Arabidopsis*. In de-etiolated pea shoots, ARF1 was concentrated mainly in the crude Golgi fractions [43]. Single loss-of-function mutants in six virtually identical ARF1 genes revealed no discernible developmental phenotypes in *Arabidopsis* [42]. Apart from cell polarity, antisense RNA studies in *Arabidopsis* have shown that ARF also affects cell expansion and cell size [44].

The expression of ARF mRNA was nearly stable throughout the different stages of suspension growth of *Arabidopsis thaliana* [45]. Kobayashi-Uehara *et al*. [46] established that the mRNA and protein levels of ARF1 were greater in root than in shoot tissue of wheat. The same expression pattern was observed in light-grown *Pisum sativum* seedlings. The level of ARF1 expression in membrane and cytosolic fractions of root tissue was higher than in shoot tissue fractions [43].

#### 2.1.2. Intra-Golgi Transport

Two different models for intra-Golgi transport were initially suggested. The direction of COPI vesicles is a critical distinguishing factor between the cisternal progression/maturation and vesicular transport models.

The vesicular transport model assumes that anterograde cargo is transported between static cisternae by coordinated budding and fusion reactions of anterograde-directed COPI vesicles [47]. Retrograde-directed COPI vesicles antagonize the continuous loss of material at the *trans*-Golgi. Thus, two different COPI vesicles would be necessary for the vesicular transport model, one mediating anterograde transport and the other mediating retrograde transport. However, the cisternal progression/maturation model does not clarify the presence of anterograde cargo within COPI vesicles or different anterograde cargo transportation rates in animal cells [48].

In the cisternal progression/maturation model, Golgi cisternae are stable compartments. Secretory cargoes are transported from one cisterna to the next in anterograde COPII vesicles, which finally disassemble at the trans-Golgi. Anterograde cargo would not leave the lumen, and resident Golgi proteins are maintained in the cisternae [47].

The exclusion of these proteins from anterograde vesicles does occur [47]. Beginning at the *cis*-Golgi, transport would be achieved by the assembly of new cisternae, which would then mature and progress along the Golgi apparatus and finally disassemble at the *trans*-Golgi. In the first model, it was demonstrated that the Golgi enzyme mannosidase II is not only present in COPI vesicles but that its concentration in these vesicles is 1.5-fold higher than in the next cisternae, as determined by quantitative immuno-electron microscopy (immuno-EM) [18]. In the second model, a biochemical approach indicated that COPI vesicles contain Golgi enzymes at a concentration that is up to 10 times higher than that found in the cisternae in animal cells [49].

The most widely accepted model for distinct and essential trafficking tasks in the Golgi is “cisternal progression/maturation”. It postulates that the stack of Golgi cisternae comprise a historical record of progression from entry at the *cis*-face to exit at the *trans*-face [50]. In this model, cargo molecules remain within a given cisternae as it passes, through an average of seven locations within the Golgi stack on its way to the *trans*-face and exit from the Golgi via transport carriers. Observations of cargo transport after a temperature block indicate a wave-like distribution that has been interpreted as cisternae with confined cargo progressing across the stack [51]. In yeast Golgi cisternae, the sequential appearance of Golgi enzymes over time has been widely cited in support of the maturation model [52]. As expected by cisternal progression, it was observed that newly arrived cargo in the Golgi exited with exponential kinetics rather than exhibiting a discrete lag or transit time [53].

Resident Golgi proteins are postulated to recycle from older to younger cisternae. Transmembrane Golgi proteins may recycle in retrograde COPI vesicles. Conserved oligomeric Golgi (COG) complex proteins facilitate the tethering of the vesicles to the target cisternae [54]. Peripheral Golgi proteins may recycle by dissociating from a given cisternae and then binding and fusing to a younger cisternae.

**Figure 2:**
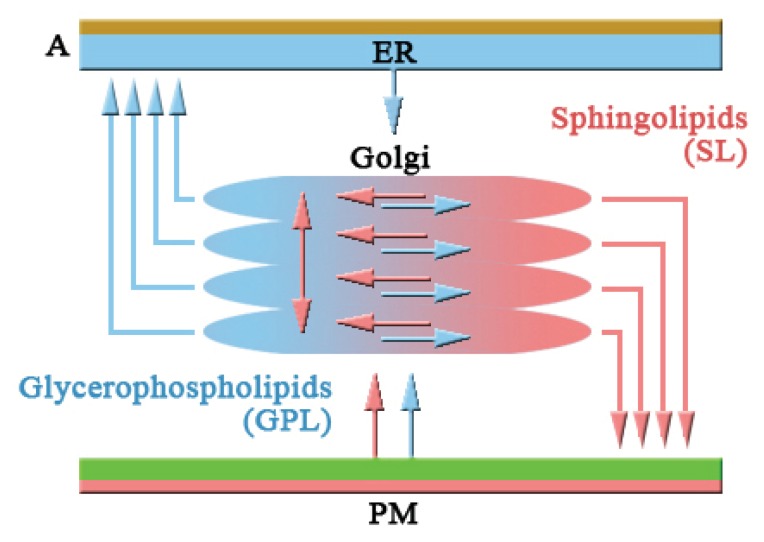

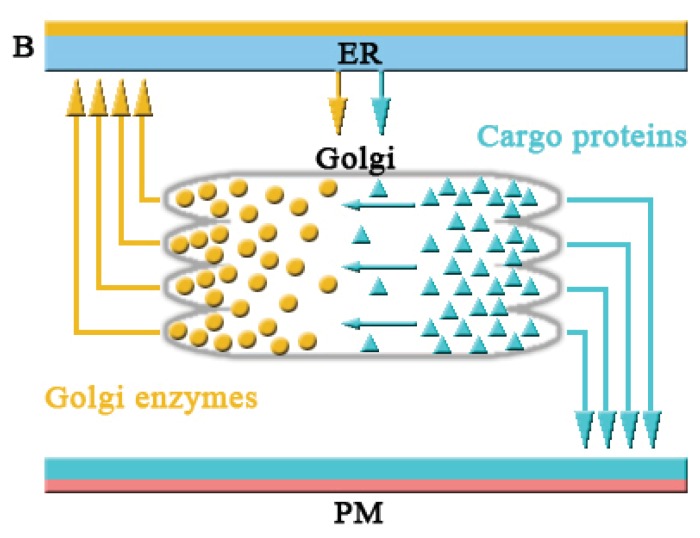
A new “*Rapid-Partitioning Model*” for intra-Golgi trafficking in mammalian system. In this model, the partitioning into domains based on the physical properties of the individual membrane components and suggests that that the Golgi membrane lipid environment consists of two components in mammalian system: Component I (Processing domain) involves glycero-phospholipids (GPL), Component II (Export Domain) involves cholesterol and glycosphingolipids (SL). (**A**) Transmembrane cargo proteins move between both lipid domains. On the other hand, Golgi enzymes are exported from export domains and move within the processing domain (**B**) [53].

Patterson *et al*. 2008 [53] proposed a new model for intra-Golgi trafficking. This model is based on partitioning of transmembrane cargo and enzymes within a two-phase membrane system: (1) Processing domain: enriched in Golgi enzymes; (2) Export domains capable of budding transport intermediates and found to clarify the full range of Golgi characteristics, including exponential cargo export kinetics, polarized lipid and protein gradients, and cargo waves. In this model, the stack-like organization of the Golgi combined with the requirement of vesicular or tubule cargo transport across it and by the segregation of lipids between two domains (processing and export) allows molecules in the system to arrange spatially (Figure 2A,B) [53].

### 2.2. SAR1 and COPII

The recruitment of COPII coat proteins involves SAR1 GTPase and its GDP/GTP exchange factor, SEC12 [9,55]. The accumulation of secretory cargo, deformation of the membrane, and formation of transport vesicles is mediated by COPII. The *Arabidopsis* genome encodes five genes for SAR1, 10 genes for the related SEC23/SEC24 proteins, and two genes each for SEC12, SEC13, and SEC31.

#### ER-to-Golgi Protein Transport

In the biosynthetic transport of proteins from the ER to the Golgi apparatus, COPII as a mediator is a milestone of early events [56].

Protein synthesis and modification occurs in the ER, and undergoes further modification, proteins leave the ER via COPII carriers to reach the Golgi. Modified proteins are sorted into the extracellular space or storage and lytic organelles from the Golgi [57]. In plants, proteins can also be sorted from the Golgi into the chloroplast [56]. This ER-to-Golgi transport is termed anterograde transport and is mediated by COPII proteins, which are highly conserved in eukaryotes [58]. Impairment of COPII function in cell systems and multicellular organisms leads to defective secretion and/or the deposition of collagen, which arises from the deficient transport of procollagen [59].

COPII assembly occurs at discrete sites on ribosome-free transitional ER (tER) or ER exit sites (ERESs) [60]. The ER membrane-associated GEF SEC12 activates the cytosolic GTPase SAR1 [61]. After activation, SAR1 associates with the ER lipid bilayer membrane, and the COPII coat composed of the SEC23-24 and SEC13-31 heterodimer complexes is recruited [62,63]. SEC23-24 and SAR1 form a cargo recruitment complex that sorts transport and ER resident proteins [64,65]. At the ERESs, SEC16 is involved in ER protein export by identifying the COPII assembly region [66]. In addition to the capture of cargo proteins, COPII is responsible for the physical deformation of the ER membrane that drives the COPII carrier formation [67,68]. GTP hydrolysis by SAR1 leads to COPII carrier uncoating. This step follows the exposure of the carrier membrane to fusion with the Golgi membrane [69].

When SAR1 protein is in the GTP-bound conformation, it binds directly to the lipid bilayer which it does by an N-terminal amphipathic alpha helix [70]. Sar1-GDP binds membranes with lower affinity [66,69]. It was shown that SAR1 lowers the mechanical rigidity of the lipid bilayer membrane to which it binds. For a vesicle-trafficking protein, it was the first discovery of membrane softening [70]. Recently, Loftus *et al*. 2012 [71] observed that Sar1p lowers the bending rigidity of the lipid bilayer to which it binds in yeast. To lower membrane rigidity ability of SAR1, they suggest a model in which membrane-bound SAR1-GTP lowers the energetic cost for the other COPII coat proteins (Sec13, Sec31, *etc*.) to generate curvature [71].

In a COPII-dependent manner, soluble cargo appears to exit the ER by bulk flow with the ER proteins efficiently retrieved from the Golgi complex [72]. In most plant cell types, similar to mammalian cells, the ER and Golgi are in close proximity, and the COPII cycles on and off the ER with a fast turnover rate [73,74]. In plants, ER-to-Golgi COPII transport might be facilitated by a Golgi scaffold that collects COPII vesicles [75]. Membrane-bound SAR1-GTP recruits the SEC23-SEC24 heterodimer by binding to the SEC23 subunit. The selection of cargo is performed by the SEC23/SEC24-SAR1 complex (prebudding complex) [76]. This complex recruits SEC13-SEC31, which provide the outer layer of the coat and drive membrane deformation to form COPII vesicles.

COPII assembly requires additional proteins, namely, SEC16 and SED4. SEC16 involves COPII coat component domains that come into direct contact and may act as a scaffold for coat assembly [77]. It was established that SEC16 contributes to the integrity of tER sites in *Pichia pastoris*. Another important protein, SED4, is an integral membrane protein located at the ER membrane. It was shown that SED4 deletion decreases the rate of ER-to-Golgi transport in *S. cerevisiae* wild-type cells [78]. Although the cytoplasmic domain of SED4 has close homology with SEC12p, no GEF activity has been reported in *S. cerevisiae* [79]. Genetic tests show that SEC12 and SED4 are not functionally interchangeable. In the same organism, SAR1, SED4, and SEC16 are hypothesized to function together during the early steps of ER vesicle assembly [78].

SEC16 has an essential role as a key organizer of ERESs in yeast and mammalian cells [80,81]. The two *Arabidopsis SEC16* genes encode for putative proteins that appear to resemble the human small isoform, which is important for ER export and tER organization in HeLa cells [81].

Previous genetic analyses have demonstrated the significance of the COPII machinery for ER-to-Golgi transport in the early secretory pathway in plants. The expression of mutant SAR1 GDP-[T34N] or GTP-[H74L] forms or overexpression of SEC12 is considered to titrate out functional SAR1 from the cytosol [72]. Export out of the ER is blocked by the retention in the ER of secretory cargo molecules or membrane proteins that cycle between the ER and Golgi apparatus [72,73,82,83]. It was suggested that there are also COPII-independent traffic pathways for protein transport from the ER [56]. However, many experimental systems revealed that COPII function is indispensable for ER-to-Golgi transport [72,73]. For example, in yeast, ER export of some proteins can proceed despite impaired Sec13 or Sec24 [67].

Three SAR1 homologs have been identified in *Arabidopsis* (AtSARA1a, AtSARA1b, and AtSARA1c). The secretion activity level from ER membranes correlated with *AtSARA1a* expression in plant cells. It was shown that blockage of ER transport to the *cis*-Golgi compartment resulted in *AtSARA1a* mRNA up-regulation [84].

The COPII coat includes four proteins, arranged as an internal receptor/cargo-binding dimer of SEC23 and SEC24, and an outer cage dimer of SEC31 and SEC13. The *Arabidopsis* genome encodes seven SEC23, three SEC24, two SEC13, and two SEC31 isoforms [85].

AtSARA1A and AtSARA1B have a 93% amino acid sequence identity but differential localization, with AtSARA1B associated with membranes to a larger extent than AtSARA1A [86]. These data suggest that different *Arabidopsis* isoforms of the same COPII elements might behave differently. Microarray analyses indicated that the COPII protein-encoding genes are ubiquitously expressed, except SAR1 (At1g09180) and a SEC31 (At1g18830) isoform [85]. Interestingly, At1g09180 not only diverges from other plant SAR1 GTPases but was also found to be expressed almost exclusively in the male organs (stamen and pollen), according to the expression profiles of COPII genes. This tissue specificity is otherwise only observed for the SEC31 isoform At1g18830, whereas all other genes appear to be ubiquitously expressed throughout the entire plant and in all developmental stages.

SAR1 was concentrated predominantly in crude ER fractions of *Pisum sativum* L. seedlings [43]. Recruitment of the COPII protein coat by SAR1p has been studied extensively [87]. In the Sar1 (A+B)-depleted cells, the prevention of COPI recruitment by inhibiting ARF activation through exposure to BFA resulted in the disassembly of the Golgi mini-stacks [56]. In addition, the Golgi mini-stacks reassembled after the removal of BFA from these cells, which indicates that all of these events were completely dependent on COPI action, regardless of the lack of COPII function [56].

*ARF1* and SAR1 proteins were several-fold more abundant in shoots relative to roots in de-etiolated pea seedlings. In total protein homogenates, the expression levels of SAR1 and *ARF1* were higher in the shoots of dark-grown pea plants than in those of light-grown plants [43].

Mammalian studies have demonstrated that the SEC24 and SAR1 isoforms have specificity for the trafficking of selective cargo in human development and disease [88,89].

**Figure 3:**
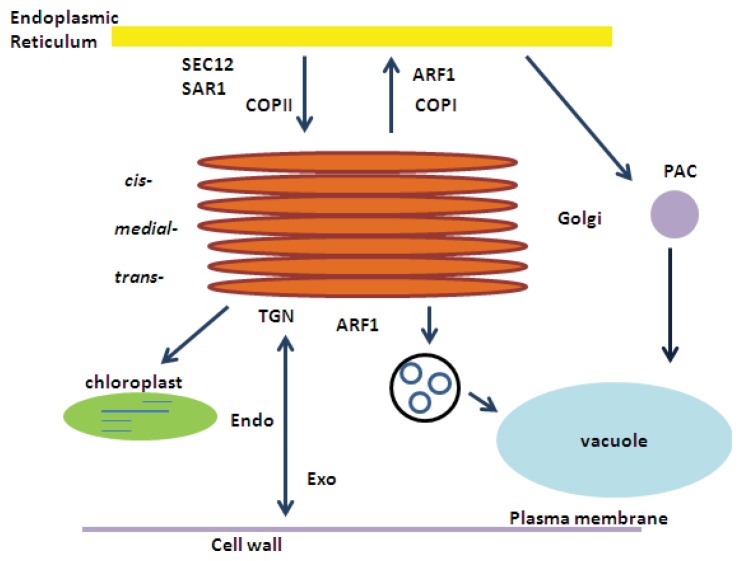
Simplified diagram of the plant endomembrane trafficking pathways. Protein synthesis and modification takes place in the endoplasmic reticulum (ER). Transportation of the proteins from the ER to the Golgi is essential for the correct protein conformation. Additional modifications and sorting of the proteins to the extracellular space-plasma membrane (PM), storage and lytic organelles is accomplished by the Golgi. Proteins can also be sorted from the Golgi to the chloroplast. The vesicles are transported to the prevacuolar compartment (PVC), which represents an intermediate compartment that is essential for proteins to reach the vacuole. The *trans*-Golgi network (TGN) is an interface between the exocytic (Exo) and endocytic (Endo) pathways. COPII proteins facilitate ER protein export, and COPI proteins control the retrograde pathway. Cargo addressed for the protein-storage vacuole is transferred from the ER to the intermediate compartment PAC (Precursor-Accumulating Compartment). The SAR1 and ARF1 GTPases are indicated in anterograde and retrograde transport, respectively (modified from [93]).

In plants, the presence of specific amino acid sequences in the primary proteins affects the selective export of membrane cargo [90]. The accumulation of SEC24A to ERESs is induced by a diacidic sequence. The interaction occurs between the K-channel KAT1, which includes the specific amino acid sequence in the cytosolic tail, and SEC24A [83,91]. Similar to mammalian cells, in plant cells SEC24 proteins bear different export signals that might lead to selective accumulation of cargo in COPII carriers. It was suggested that more efficient intracellular trafficking is likely accomplished by cargo specialization of COPII isoforms in multicellular organisms [92]. Figure 3 shows the simplified diagram of the retrograde and anterograde transport in plant cells.

## 3. Developmental Regulation of ARF1 and SAR1

Correct protein localization and maintenance of membrane homeostasis is achieved by the secretory pathway. This pathway is also essential for the secretion of extracellular factors that are involved in developmental processes.

In the secretory pathway, proteins intended for the cell surface or other organelles are incorporated into vesicles that bud from the ER membranes. For all of these budding events, the COPII protein coat may be involved by providing the means for membrane deformation, as well as for the integration of integral membrane cargo into budding vesicles [9,56].

To conduct a functional analysis of small GTPases, embryogenesis, cell plate formation, and cellular polar growth, which occur in the pollen tubes and root hairs, are the most common biological systems.

Cell polarity is crucial for the development of most eukaryotes. In many biological processes, such as polarity development and the regulation of plant responses to environmental stresses, small GTPases play key regulatory roles [94–96]. Root hairs and pollen tubes have amplified turnover rates of cytoskeletal proteins, cytoplasmic structures, and organelles. Membrane and cell wall materials deliver to the growing tip of the root through increased membrane vesicle trafficking [96].

The distribution of coated vesicles along the tip of the root hair varied with both age and growth rate [97,98]. During the active growth stage, coated vesicles emerge from the subapical plasma membrane in the clear zone; however, the incidence of these events decreases when the root hairs reach maturity [96]. In *Pisum sativum* L. seedlings, *ARF1* protein levels did not fluctuate significantly in the root and shoot tissue during early development [43].

SAR1 is regulated at both the transcriptional and translational levels. SAR1 protein expression was increased during the development of root tissue. However, in the same subcellular protein fractions of shoot tissues, a decreasing SAR1 accumulation pattern was detected. It is likely that the general decrease in SAR1 in mature shoots reflects reduced secretory activity compared with immature shoots. The SAR1 protein expression pattern suggests a possible role of this gene in early development and polar growth [43].

During early developmental stages, the relative abundance of SAR1 protein in the root tissues suggests a high level of Golgi-to-ER vesicular transport. Interestingly, ARF1 protein expression was reduced and SAR1 expression increased in root tissue. qRT-PCR analyses indicated that the level of ARF1 mRNA was approximately the same at all developmental stages.

## 4. Light Regulation of ARF1 and SAR1

The effect of light on plant development was elegantly demonstrated by the light control of early seedling development. Seedlings grown in the dark follow a skotomophogenic developmental process and display an etiolated phenotype characterized by their elongated hypocotyls and folded cotyledons with apical hooks. In contrast, seedlings grown in the light follow a photomorphogenic developmental process and display a de-etiolated phenotype, which includes the inhibition of hypocotyl elongation, unfolding of apical hooks, expansion of cotyledons, and expression of light-regulated genes. After exposure to light, the cotyledons open and expand, and stem elongation slows dramatically [99]. These events are referred to as photomorphogenesis. During the life cycle of a plant, light quality, duration, and intensity have a serious affect on plant development. Small GTPases perform important roles in light signal transduction in plants [100]. However, the relationship of these proteins with regard to the light signal transduction pathway regulation is not fully understood. The pea Pra2 small G protein, a YTP/RAB family member, is unique because its molecular mechanism for light-regulated expression has been characterized in detail [101,102]. The Pra2 protein participates in vesicle transport occurring in stem elongation of etiolated seedlings [100]. Pra2 activity has been reported to be suppressed by light and induced by dark in pea seedlings [99,101].

Because light is a potent regulator of plant growth and development, the regulation of ARF1 and SAR1 by white light was investigated. Regulation by light implies that SAR1 may have a developmental function in plants. Out of all the developmental phases, seedling development is the most sensitive to light [103,104]. The regulation of SAR1 by light at the mRNA transcript level demonstrates that this small GTPase may have a developmental role in plants [43].

Cellular growth, daily changes in photosynthetic activities, and leaflet movement are controlled by the circadian clock in plants. Little is known regarding the regulation of ARF1 and SAR1 in plant circadian systems. In the etiolated shoots of pea seedlings, the SAR1 mRNA expression level exhibited circadian rhythmicity in qRT-PCR experiments.

## 5. Conclusions and Perspectives

ARF1 has been shown to have a significant role in COPI-mediated retrograde trafficking in eukaryotic systems [9,53,105–108]. mRNA expression analyses have indicated that ARF levels are nearly constant throughout the different stages of suspension growth of *A. thaliana* [45]. Kobayashi-Uehara *et al*. [46] demonstrated that the mRNA and protein levels of ARF1 were greater in the root tissue than in the shoot tissue of wheat. Our results corroborate this finding, as ARF1 protein levels in both cellular fractions (membrane and cytosolic fractions) of the root are higher than in the shoot tissue of *P. sativum*. During the early development stage, the ARF1 mRNA expression profile data suggest that ARF1 is regulated at the transcriptional level rather than at the translational level in pea shoot tissue.

The other important small GTP binding protein SAR1 is involved in the intracellular COPII-mediated protein trafficking from the endoplasmic reticulum to Golgi apparatus [9,53]. Bioinformatics analyses have established SAR1 homologues in various plant species, including *A. thaliana*, *Nicotiana tabacum*, and *Brassica campestris* [2,109,110]. At least one of the tobacco and one of the *Arabidopsis* SAR1 isoforms are involved in ER export [72,73,111]. This gene is regulated at both the transcriptional and translational levels in our experiments with *Pisum sativum*. Our data demonstrate that a general reduction in SAR1 in mature shoots may reflect lower secretory activity compared with young shoots. The SAR1 protein expression pattern suggests a possible role for this small GTPase in early development and polar growth.

Proteomic and transcriptomic approaches have opened up new and promising research areas for these small GTPases. In particular, genome-wide quantification of gene expression with “Next Generation Sequencing” (NGS) technology will identify the components of endomembrane trafficking in different plant cells.
